# Nursing care as a systemic and entrepreneurial phenomenon

**DOI:** 10.1590/1980-220X-REEUSP-2022-0249en

**Published:** 2022-09-23

**Authors:** Dirce Stein Backes, Mateus Claudio Zinhani, Alacoque Lorenzini Erdmann, Marli Terezinha Stein Backes, Andreas Büscher, Mara Regina Teixeira Caino Marchiori

**Affiliations:** 1 Universidade Franciscana, Santa Maria, RS, Brazil.; 2 Universidade Federal de Santa Catarina, Florianópolis, SC, Brazil.; 3 Hochschule Osnabrück, Osnabrück, Germany.

**Keywords:** Nursing Care, Leadership, Pandemics, Nurse's Roles, Public Health Nursing, Nonlinear Dynamics, Atención de Enfermería, Liderazgo, Pandemias, Rol de la Enfermera, Enfermería en Salud Pública, Dinámicas no Lineales, Cuidados de Enfermagem, Liderança, Pandemia, Papel do Profissional de Enfermagem, Enfermagem em Saúde Pública, Dinâmica não Linear

## Abstract

The objective is to produce a critical-reflexivity analysis of nursing care, from the perspective of complexity thinking and social entrepreneurship. Theoretical-reflective study, supported by the framework of complexity thinking and social entrepreneurship. The main characteristics that lead and support nursing care are analyzed from a systemic-entrepreneurial perspective. A parallel is conceived between vertical care, design from a hierarchical structure and nursing care in the systemic-entrepreneurial perspective, which leads to singularity, originality, circularity, complementarity and interactivity. The centrality of nursing care is reaffirmed as a tangible social good or not. Theoretical reflection on nursing care as a systemic and entrepreneurial phenomenon raises a unique and multidimensional perception of the human being/user, health, the nursing work process, in order to achieve an increasingly agile, dynamic, circular, complementary and interdependent care.

## RESUMEN

Este estudio pretende realizar un análisis crítico-reflexivo de los cuidados de enfermería desde la perspectiva del pensamiento de la complejidad y del emprendimiento social. Se trata de un estudio teórico-reflexivo, apoyado en el marco del pensamiento de la complejidad y del emprendimiento social. Se analizan las principales características que conducen y sostienen los cuidados de enfermería desde una perspectiva sistémico-emprendedora. Se establece un paralelismo entre los cuidados verticales, perfilados desde una estructura jerárquica, y los cuidados de enfermería, desde una perspectiva sistémico-emprendedora, que conduce a la singularidad, la originalidad, la circularidad, la complementariedad y la interactividad. Se reafirma la centralidad de los cuidados de enfermería como bien social tangible o intangible. La reflexión teórica sobre el cuidado enfermero como fenómeno sistémico y emprendedor plantea una percepción singular y multidimensional del ser humano/usuario, de la salud y del proceso de trabajo enfermero, para lograr un cuidado cada vez más ágil, dinámico, circular, complementario e interdependiente.

## INTRODUCTION

This study originated from the question: what is new in nursing care and how did it differ in the pandemic period? It was noted, without great premeditation, that nursing care is original, innovative, transformative and always entrepreneurial. Based on this, the Nurse will always be an entrepreneur and care, consequently, will always have an aggregating effect on well-being and social value, whether tangible or not, considered one of the main characteristics of social entrepreneurship^(1)^.

Under this impulse, nursing care can/should be characterized as a systemic and entrepreneurial phenomenon due to its unique, original and transformative character. Stimulated through multiple relationships, interactions and systemic associations, nursing care always has the well-being of the person at an individual and collective level as its ultimate purpose^(2)^. Therefore, nursing care transcends the punctual and vertical perspective of being and moving only as an action and encompasses a movement of circular, complementary and dialogic dialogue between actors – caregiver and person/family/community under care^(3)^.

Therefore, nursing care must be understood and promoted as a social investment, capable of generating comfort, relief and well-being^(4)^. In its innovative dimension, aggregating and enhancing life and health, nursing care constitutes, by excellence, an important contribution to people’s quality of life. However, there will be no innovative and transformative care without entrepreneurial leaders nurses^(5,6)^.

The concept of entrepreneurship has been widely discussed in different areas of knowledge. While some portray entrepreneurship out of necessity or opportunity, others focus on social entrepreneurship that aims to promote social, cultural, economic and environmental quality of life from the perspective of sustainability(7,8,9). In addition to these conceptions, nursing entrepreneurship may be related to the quality of life, the values and principles of a post-pandemic sense of life and human living^(10)^.

Therefore, nursing plays an important role in health promotion, protection and education, as well as in curative care, in consulting and advisory projects, among others. Since the beginning of the Covid-19 pandemic, several studies have addressed the role of nursing and have shown that these professionals, regardless of the circumstances, have developed a great sense of social responsibility, in addition to playing a leading role in various health care processes^(11,12)^. However, few of them sought to uncover the real impact of nursing care in the community and how it differs from other professional knowledge and practices^(13,14,15)^.

Based on the above, the objective is to produce a critical- reflexivity analysis of nursing care, from the perspective of complexity thinking and social entrepreneurship.

## METHOD

Theoretical-reflective study, supported by the systemic framework and social entrepreneurship. The main characteristics that lead and support nursing care are analyzed from a systemic- entrepreneurial perspective. Complex systemic thinking and social entrepreneurship are established as relevant references for understanding what users increasingly expect and need from nursing/health professionals – a singular and multidimensional care.

Morin^(16)^, the protagonist of complexity thinking, did not foresee a predefined methodological path to analyze and describe social phenomena. It encourages a path of its own, in which the researcher is induced, as a protagonist, to learn, invent and modify his itinerary based on the (re)construction and expansion of his own knowledge.

This study is designed in this direction from references that materialize when inventing, questioning, and weaving together the experiences lived in learning, teaching, investigating, bringing together and in health and nursing care. Therefore, a schematic parallel between traditional vertical care, conceived from a linear and punctual hierarchical structure, and nursing care from a systemic-entrepreneurial perspective is made possible.

Thus, the theoretical-reflexivity framework consists of productions by Edgar Morin, which preserve the core of systemic-complex, above all, evolutionary and transformative thinking^(16–18)^, in addition to productions that support nursing care as an entrepreneur^(19,20,21)^. So, concepts such as singularity, multidimensionality, originality, interactivity, complementarity and transformation will be explored, without giving them as conclusive.

## FROM THE VERTICAL HIERARCHICAL CONCEPTION TO THE SYSTEMIC-ENTREPRENEURIAL PERSPECTIVE OF CARE

In the different health services, surveys are carried out that assess user satisfaction in relation to their expectations, suggestions and feedback on nursing care, in order to then develop strategies to improve quality. However, the focus of this study is not reduced to the analysis of the quality of nursing care, but to demonstrate how much it is and can, increasingly, be original, innovative and transformative, considering that each user is unique and demanding of care that addresses specific needs.

**Figure 1.**
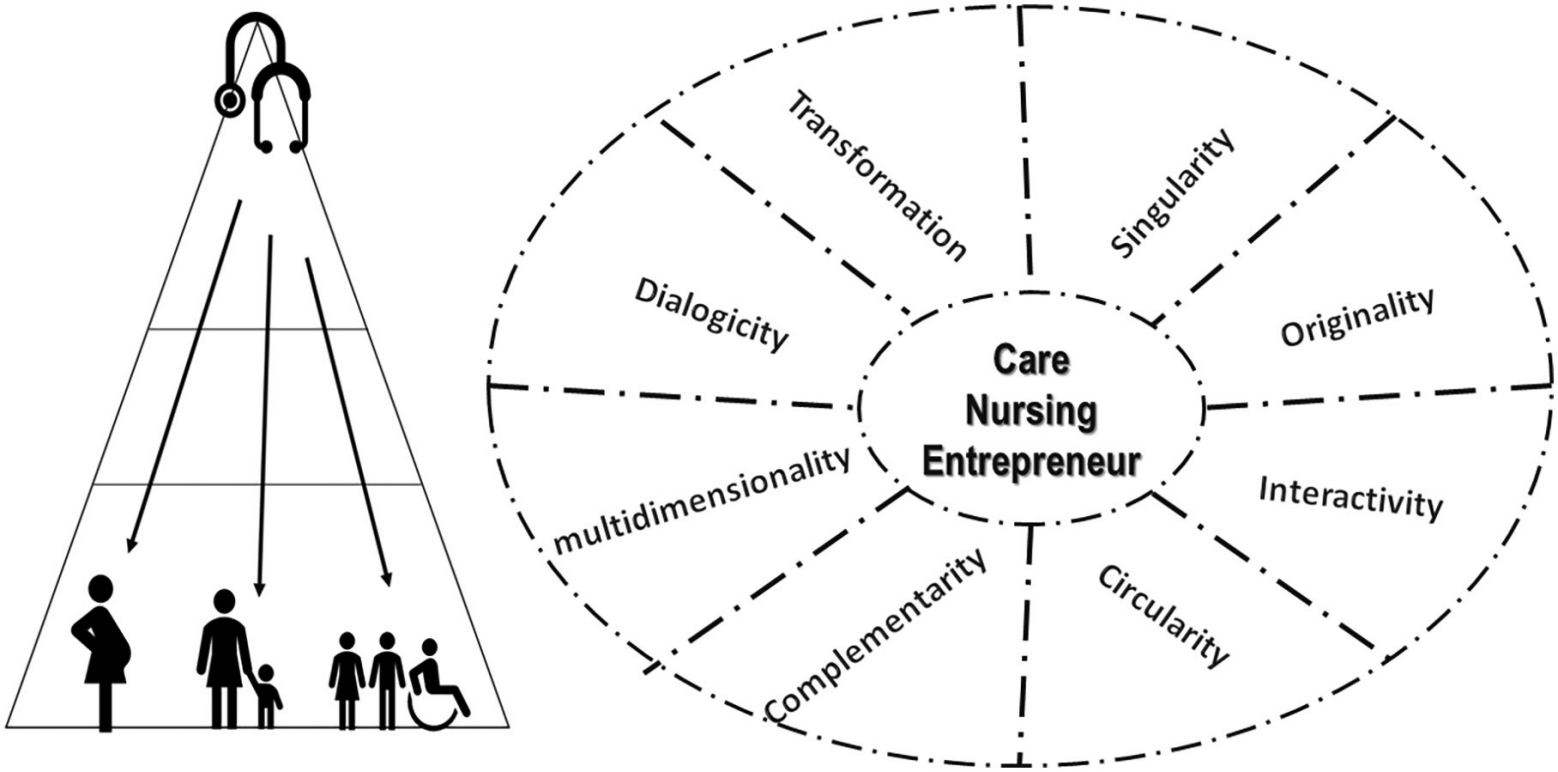
Vertical hierarchical approach and the systemic-entrepreneurial perspective of nursing care – Santa Maria, RS, Brazil, 2022.

Next, a schematic parallel is presented between vertical care, designed from a hierarchical structure, and nursing care in the systemic-entrepreneurial perspective, which leads to singularity, originality, interactivity, circularity and complementarity. The centrality of nursing care as social welfare is reaffirmed. The predominant characteristics in each of the approaches are shown and it is suggested not the rupture of the vertical hierarchical structure, but its evolution in the sense of reaching increasingly higher and advanced levels in relation to health care, as shown in Figure 1.

The theoretical-reflective description is conducted, based on the proposed schematic parallel, through the design of two exploratory categories: Hierarchical vertical structure of care – punctual and linear actions; and Systemic-entrepreneurial perspective of care – from linearity to transforming circular interactivity.

## HIERARCHICAL VERTICAL STRUCTURE OF CARE – PUNCTUAL AND LINEAR ACTIONS

The vertical structure has as predominant characteristics the order and centralization of decisions by the health professional. In this logic, vertical interaction between professional and user/family/community prevails, rigid control mechanisms and greater trust in protocols, flowcharts, pre-established routines, among other regulations. Users, who receive care, generally do not participate in decisions and, consequently, are not encouraged to develop their autonomy, empowerment and self-care. In addition, the health user is determined by the unilateral and disciplinary order, while the health professional assumes, in most cases, authoritarian characteristics, demanded by the hegemony of the power-to-do.

Even in this hierarchical logic, the technical, punctual, linear and decontextualized knowledge about professional-user interactions prevails, with greater interest in maintaining hegemonic power than in discussing/negotiating specific demands and needs of users. Users are rarely heard and welcomed in their real health needs and even more rarely are they encouraged to develop their role in self-care.

The hierarchical vertical logic offers an advantage to punctual decisions, sometimes necessary to direct and control the nursing work process in an agile and interventionist or assistance way. This structure demands professional attributions based on technical-scientific knowledge to maintain life and avoid death. However, this path, usually routine and protocol, easily incurs in the depersonalization of care and, consequently, in accommodation, disinterest and dehumanization of care.

The vertical, fractional and highly specialized concept has become, from the systemic-entrepreneurial perspective, insufficient to respond to the growing complexity of health care. It is necessary to have, in addition to nurses with specific and highly specialized functions, leaders able to make collegiate and interprofessional decisions, capable of valuing and enhancing initiatives and knowledge of users. Advanced nursing practice, user satisfaction and the quality of care depend greatly on Nursing leadership skills and competences^(22)^. 

It urges the need to advance in the direction of systemic- complex-entrepreneurial thinking. Although it is more comfortable to maintain routine, order, control and linear and highly specialized actions, it is essential to follow the evolutionary dynamics of the systems, which has worsened even more with the Covid-19 pandemic. For Morin, a strictly deterministic and specialized social system, demanding only order, would be a universe without innovation, without transforming prospects^(16,17)^. However, how to overcome the deterministic pyramidal structure in order to intuit prospective circular movements that contemplate, in essence, singularity, multidimensionality, originality, interactivity, complementarity and transformation by nursing care?

## SYSTEMIC-ENTREPRENEURIAL PERSPECTIVE OF NURSING CARE – FROM LINEARITY TO TRANSFORMING CIRCULAR INTERACTIVITY

In the systemic-entrepreneurial logic of care, functional arrangements are flexible, dynamic, complementary and interdependent. The professional user/family/community horizontal interaction prevails over the hierarchical vertical logic. Professional functions are (re)defined in the interaction between the various actors involved in the care process. Nursing care, from this point of view, must be understood as what is woven together, in association and interdependence with all other health professionals and users. Therefore, any noise or evolution in one of the weaving threads simultaneously affects the complex unit – care in its singular and multidimensional dimension.

Promoting care in this systemic-entrepreneurial perspective implies living with different social actors, dialoguing in different situations, reinventing during uncertainties and (re)building oneself throughout life. It implies the ability to integrate the notions of order and disorder, deal with conflicts and readjust to the continuously changing conditions of the environment^(16)^.

Based on this premise, the following aspects show that nursing care is a systemic and entrepreneurial phenomenon, insofar as:

– It enables reception without barriers, prejudices or personal interests and establishes empathic relationships, demonstrating that each user is worthy of dignified, respectful and affable care.
– It enables active/meaningful listening and manages to extract the best in each user, in order to make them even more special.
– It transcends prescriptive protocols/recipes and manages to pay attention to the uniqueness of each user/family/community.
– It promotes the well-being of the user and family, by relieving pain and tension, anguish, doubts and uncertainties.
– It satisfies needs expressed and/or not, through interactions and the trust bond.
– It encourages horizontal, dialogic and synergistic exchanges that favor healthy living.
– It provides an aggregating and stimulating environment for new thinking.
– It reinforces initiatives, expands possibilities and enhances the potential of the user/family, in addition to valuing each user’s achievement and demonstrates that he is the main agent of change.
– It promotes health care policies in all segments of society, based on a collaborative network in favor of best practices for demands, bottlenecks and vulnerable situations of health care needs.
– It prospects new modalities and environments for professional-user dialogue, such as the Telenursing, health education on YouTube, among others.
– It protagonizes forward-looking social movements of nursing and health care, by overcoming barriers, opening new niches of genuine cultures of health care.

In order to reach higher and more advanced levels in relation to nursing care, it is essential to transcend disciplinary barriers and achieve an integrated and articulated knowledge with different areas. This process implies (de)constructing professional knowledge and practices, overcoming theoretical reductionism and prospecting strategies that value authority and leadership to the detriment of the hegemonic prescriptive order.

## NURSING CARE AS A SYSTEMIC AND ENTREPRENEURIAL PHENOMENON

The understanding of nursing care is related to a complex of elements in mutual, evolutionary and transforming interaction. Like other systems, nursing care is subsidized by subsystems and, at the same time, is part of a larger system – the Unified Health System, which interacts with other social systems. Systemic-entrepreneurial care moves and feeds back from circular and interdependent movements between users, professionals, services, communities, and the social system. The change in a subsystem (re)produces itself with the larger system, according to its own evolutionary and transforming dynamics^(16–18)^.

Based on this approach, the quality and impact of nursing care is determined by the quality of dialogic, visionary and prospective relationships, interactions and associations with the different actors involved in the health care process^(23)^. A study reinforces this thinking, by mentioning that in addition to the quality of interactions between professional-users, care is determined by the reception and attention to users’ needs and respect for their dignity^(24)^.

In this context, the quality and impact of nursing care influences and is influenced by health indicators, in general, of individuals, families and communities and, in the same way, is related to morbidity and mortality rates, which consequently can be associated with poor quality of care. Although the causes of death are, in most cases, multifactorial and strongly linked to economic and health conditions, they are highly dependent on sensitive and relational indicators that indicate the quality of health care, especially nursing professionals^(25,26)^.

Sensitive indicators may be associated and, in some cases, determined by working conditions and the health care process. A study shows that health professionals and users report greater satisfaction with health care, as they show that it generates well-being and adds social value^(1)^. Therefore, entrepreneurial nursing care has a direct relationship with the meaning of work, ambience, welcoming, openness to the new and social commitment. Thus, the mediator, in this case the enterprising nurse, has a relevant role in prospectively leading the care process, based on horizontal and dialogic technologies^(27)^.

However, it is not enough just to strengthen the leadership of the Nurse – goal of the Nursing Now campaign^(28)^. In addition to this goal, it is necessary to develop the thinking of complexity and intuit an entrepreneurial behavior both in teaching and research, as well as in the apprehension and dynamization of nursing care, with a view to the (re)organization, expansion and prospection of nursing care as a systemic and entrepreneurial phenomenon.

The Covid-19 pandemic generated unprecedented tensions and exhaustion among the nursing team, but it also allowed for advancement, achievements and (re) constructions. Although nurses are considered the most reliable profession, the level of their influence in functions and decision-making positions does not correspond to public recognition^(29)^. The pandemic period led professionals, in general, to (re)organize their systems and review their theories and practices, based on references that expand, contextualize and consider both the uniqueness and the human multidimensionality of care and health.

The pandemic exacerbated the relevance of nursing care in the different environments of human-social dialogue, in addition to proving that nursing does not have a collection of absolute, lasting and unquestionable truths. It is opportune to apprehend the lessons and lessons learned from it, in addition to taking advantage of the moment to (re)signify attitudes, postures, professional values and enhance nursing care as a systemic and entrepreneurial phenomenon.

Conceiving nursing care as a systemic and entrepreneurial phenomenon necessarily implies expanding the concepts of human being, life, health, environment and time – present and future. If the nursing professional has the skills and potential to produce care characterized as common-social good, he also has the ability to evolve, (re)build, aggregate, innovate and prospect new health strategies and policies.

The contributions of this study to the advancement of nursing science are related to the enhancement of nursing care as a systemic and entrepreneurial phenomenon and, of the Nurse, as a mediator of increasingly agile, dynamic, circular, complementary and interdependent care processes. Another contribution is associated with the promotion of a new thinking among nursing professionals, based on references that expand and prospect possibilities and new niches of action, both in the social, political and economic spheres.

It is considered, as a limitation of this study, the proposition of only two theoretical-practical references – systemic-complex and entrepreneurship, when there are many others with the potential to leverage nursing care as social welfare in evolution. However, it is hoped that other thinkers advance in this theoretical proposition and that they contribute to enhancing and positioning care in the desired social understanding and recognition.

## FINAL CONSIDERATIONS

Theoretical reflection on nursing care as a systemic and entrepreneurial phenomenon raises a unique and multidimensional perception of the human being/user, health, the nursing work process, in order to achieve an increasingly agile, dynamic, circular, complementary and interdependent.

The Covid-19 pandemic has generated tensions and negative repercussions in almost all social systems. But for nursing, in particular, this moment revealed its impact on health care and the opportunity to enhance nursing care as a sensitive, evolutionary, lasting, non-negotiable and priceless social good, therefore, entrepreneur.
